# UV-Enhanced Ethanol Sensing Properties of RF Magnetron-Sputtered ZnO Film

**DOI:** 10.3390/s18010050

**Published:** 2017-12-26

**Authors:** Jinyu Huang, Yu Du, Quan Wang, Hao Zhang, Youfu Geng, Xuejin Li, Xiaoqing Tian

**Affiliations:** Shenzhen Key Laboratory of Sensor Technology, College of Physics and Energy, Shenzhen University, Shenzhen 518060, China; jinyu8669@126.com (J.H.); wq13575733860@126.com (Q.W.); haozhang@szu.edu.cn (H.Z.); gengyf@szu.edu.cn (Y.G.); lixuejin@szu.edu.cn (X.L.)

**Keywords:** ZnO film, UV illumination, magnetron sputtering, ethanol sensing properties

## Abstract

ZnO film was deposited by the magnetron sputtering method. The thickness of ZnO film is approximately 2 μm. The influence of UV light illumination on C_2_H_5_OH sensing properties of ZnO film was investigated. Gas sensing results revealed that the UV-illuminated ZnO film displays excellent C_2_H_5_OH characteristics in terms of high sensitivity, excellent selectivity, rapid response/recovery, and low detection limit down to 0.1 ppm. The excellent sensing performance of the sensor with UV activation could be attributed to the photocatalytic oxidation of ethanol on the surface of the ZnO film, the planar film structure with high utilizing efficiency of UV light, high electron mobility, and a good surface/volume ratio of of ZnO film with a relatively rough and porous surface.

## 1. Introduction

Zinc oxide (ZnO) is a one of the wide band-gap metal oxide semiconductors and, as a promising candidate for gas sensors, has been widely applied in detecting ethanol and other various harmful gases [1,2,3,4]. The response mechanism of ZnO is that the resistance changes dramatically when the target gas reacts with the chemically-absorbed oxygen of the zinc oxide [5]. In general, in order to speed up the response rate and overcome the activation energy of the reaction, the operating temperature of semiconducting-oxide gas sensors is very high (2000 °C). However, this high temperature is not conducive to reducing power consumption, and makes the sensor difficult for usage in integrated circuits. In addition, the secondary growth and aggregation of metal oxide particles at high temperatures may lead to undesirable long-term drift problems.

In recent years, many techniques, such as doping of noble metals [6,7], MEMS fabrication [8], nanosensing materials [9], usage of electrostatic fields [10], and ultraviolet (UV) irradiation [11,12], have been adopted to reduce the operating temperature of these sensors. Among them, as a promising strategy, UV light irradiation has attracted a great deal of attention. A few reports have indicated that the sensing performances of semiconducting oxide gas sensors could be improved noticeably under UV light irradiation [13,14,15,16]. However, most studies focus on the design and synthesis of novel sensing materials with various morphologies and microstructure. To date, the reported gas sensors with UV illumination usually utilized a tubular structure, and most of the sensing materials on the backside of the tubular-structure sensor cannot be illuminated, which led to an insufficient influence of UV illumination on the sensing performance. The film structure, having high electron mobility and high utilizing efficiency of UV light, is proposed as a promising material for UV-enhanced gas sensing applications. However, the planar-type film gas sensors with UV illumination have been rarely proposed [17,18,19].

Up to now, many methods have been used to produce ZnO thin films. Among them, the magnetron sputtering method has demonstrated a great deal of attractive advantages, such as long-term stability and well-controlled sputtering process, high deposition rates, and good crystallization of the deposited film [20,21,22]. Thus, in this work, a planar-type gas sensor based on ZnO film has been deposited by the magnetron sputtering method. The gas sensing performance of the planar-type sensor with and without UV illumination was investigated.

## 2. Experimental

### 2.1. Preparation of the ZnO Film

A magnetron sputtering system was used for deposition of ZnO film. ZnO (99.9%) employed as the sputtering target was sputtered in a commercial alumina substrate pre-printed with the interdigitated gold electrodes. The chamber was evacuated to the base pressure of about 10^−5^ Pa, and the deposition pressure was maintained at 308 Pa. The sputtering power was 120 W and the time of RF application was about 100 min. The deposited ZnO layers were annealed at temperature of 450 °C for 60 min.

### 2.2. Characterizations

The crystalline structure of the film was characterized by X-ray diffraction (XRD, Rigaku, Tokyo, Japan). Morphological property of the deposited sample was investigated by Scanning electron microscopy (SEM, FBI Nova S-450, FEI, New York, NY, USA). Transmission electron microscopy (TEM) images were recorded on a FEI Tecnai G220 (New York, NY, USA) transmission electron microscope under a working voltage of 200 kV. The gas-sensing tests were carried out on a commercial CGS-4TPs gas sensing analysis system (Beijing Elite Tech Co., Ltd., Beijing, China.)

### 2.3. Fabrication and Gas Sensing Measurements

**Figure 1:**
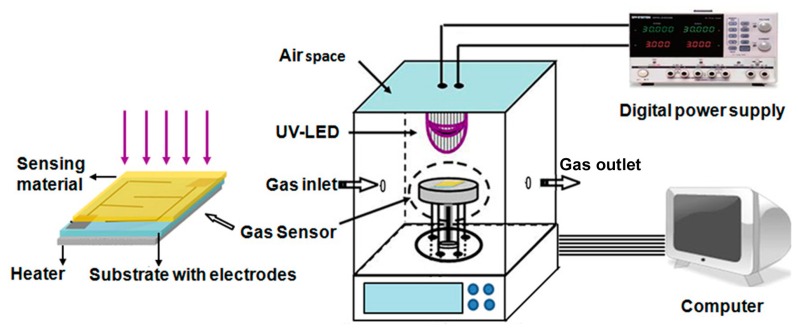
The schematic structure of the gas sensor and gas sensing measurement system.

Gas sensing measurement on the as-deposited ZnO thin film sensors were carried out by using a climate chamber (Figure 1). A UV-LED light (365 nm; 0.5W/cm^2^) was used as the light source of the UV-illuminated sensor. The distance between the UV-LED light source and gas sensors was kept around 7 cm. The sensor was first irradiated with UV-LED light for 30 min to stabilize their electrical properties. The response of the gas sensor is defined as the ratio of the resistance of the sensor in dry air (R_a_) to that in the target gases (R_g_). The response or recovery time was defined as the time taken by the sensor resistance output to reach 90% of its total resistance change after each process of applying or clearing the target gas.

## 3. Results and Discussion

### 3.1. Structure and Morphology of the As-Prepared Material

**Figure 2:**
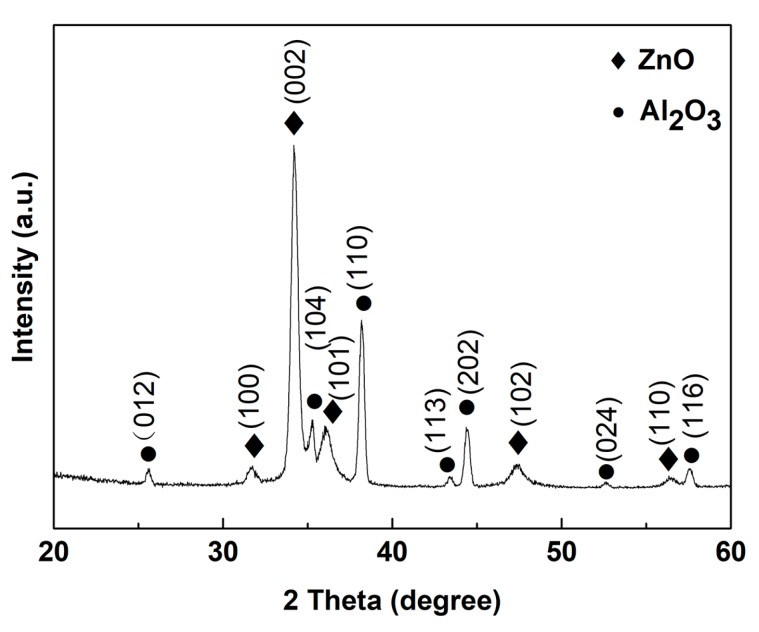
XRD pattern of the synthesized ZnO film deposited by RF magnetron sputtering.

The XRD pattern of the ZnO thin film is illustrated in Figure 2. The main XRD peaks positioned at 31.6°, 34.2°, 36.1°, 47.3°, and 56.5° were assigned to the scattering from (100), (002), (101), (102), and (110) planes of wurtzite-type hexagonal ZnO (JCPDS no. 36-1451) along with a preferentially c-axis orientation. On the other hand, the low reflection peaks observed at 25.5°, 35.2°, 38.2°, 43.5°, 44.3°, 52.6°, and 57.5° were assigned to the (102), (104), (110), (113), (202), (024), and (116) planes of alumina (Al_2_O_3_) substrate. No other peaks belonging to impurities were observed in the XRD pattern suggesting the successful growth of crystalline ZnO film on the Al_2_O_3_ substrate.

**Figure 3:**
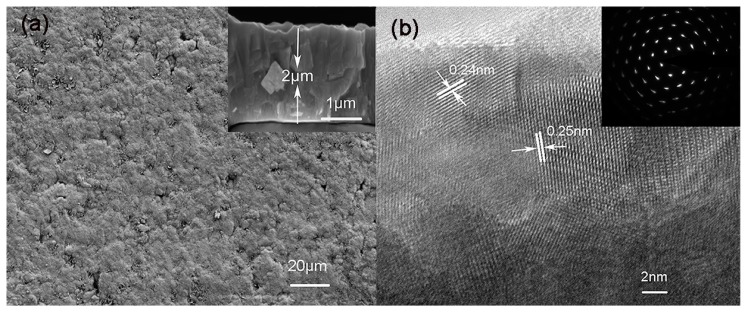
(**a**) SEM images of ZnO film; and (**b**) HRTEM and SAED images of ZnO film.

The morphology and structure of the as-deposited ZnO film were characterized by SEM and HRTEM (High resolution-transmission electron microscopy). The SEM image (Figure 3) shows that the surface of as-deposited ZnO film is relatively rough and has a porous structure. Additionally, the thickness of ZnO film is approximately 2 μm based on the cross-section image (shown in the inset of Figure 3a). The selected area diffraction (SAED) pattern and HRTEM images taken from a random particle on ZnO film are shown in Figure 3b. Ordered lattice fringes were clearly seen from the HRTEM image of ZnO film. The lattice spacing are 0.25 nm and 0.24 nm, corresponding to the (002) and (101) planes of the wurtzite ZnO, respectively. The SAED pattern of ZnO film (shown in the inset of Figure 3b) displays several ordered bright diffraction spots, suggesting the single crystalline behavior of the as-deposited film.

### 3.2. Sensing Properties

**Figure 4:**
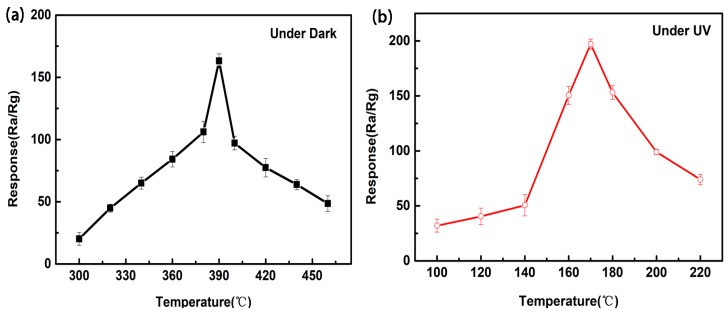
Response of the ZnO film sensor to 1000 ppm ethanol (**a**) under dark and (**b**) under UV light illumination as a function of the operating temperature.

Gas sensing properties of the as-deposited ZnO film were studied towards C_2_H_5_OH. The responses of the sensor to a fixed 1000 ppm concentration of C_2_H_5_OH were measured at different temperatures and the observed results are presented in Figure 4a,b. The maximum response of the sensor without UV light illumination is 163 at 390 °C, while the response reached to 197 at 170 °C under UV illumination. Noticeably, the UV-illuminated ZnO-based sensor exhibits much lower optimum sensing temperature and higher response compared to that of without UV illumination.

**Figure 5:**
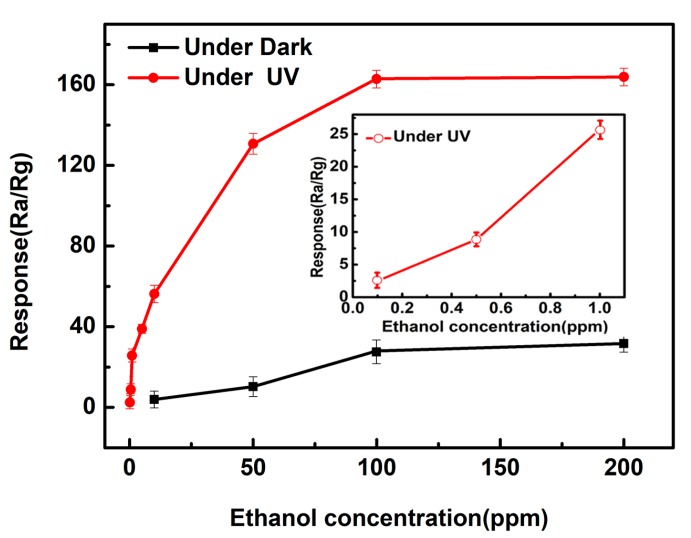
Response of the ZnO film sensor to different concentrations of C_2_H_5_OH from 0.1 to 200 ppm under dark and under UV LED activation.

The plot of the response as a function of different C_2_H_5_OH concentrations of the ZnO film sensor with and without UV light illumination at their optimum operating temperatures is depicted in Figure 5. Compared to without UV illumination, the response of the UV-illuminated ZnO sensor increases more rapidly with the increase of C_2_H_5_OH concentration below 100 ppm. Afterwards (above 100 ppm), the sensors tended to saturate gradually. It is noted that the sensor with UV light illumination exhibits an obvious response to C_2_H_5_OH concentration even as low as 0.1 ppm (response = 2.5).

**Figure 6:**
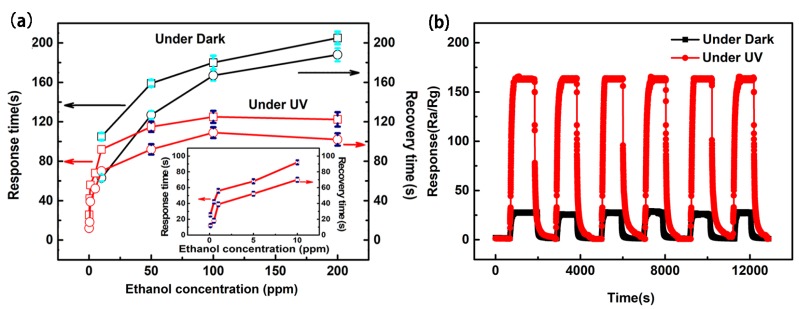
(**a**) The response and recovery times to different concentrations of C_2_H_5_OH and (**b**) six cycles of response curves to 100 ppm C_2_H_5_OH of the ZnO film sensors under dark and under UV light illumination.

The response and recovery characteristics of the ZnO film sensor to different concentrations of C_2_H_5_OH with and without UV light illumination at their optimum operating temperatures were also studied (Figure 6a), wherein the response and recovery times of the sensor to 100 ppm C_2_H_5_OH with UV light illumination are about 125 s and 109 s, respectively. Comparatively, those of the sensor without UV light illumination are approximately 180 s and 171 s, respectively. The sensor with UV light illumination exhibits a more rapid response–recovery process to C_2_H_5_OH than that under dark conditions. Reproducibility measurements on the ZnO film sensor (with and without UV illumination) were also carried out on a fixed 100 ppm C_2_H_5_OH concentration and the subsequent results are displayed in Figure 6b. The observed six reversible cycles of the ZnO response curves indicate that both of the sensors have good reproducibility.

**Figure 7:**
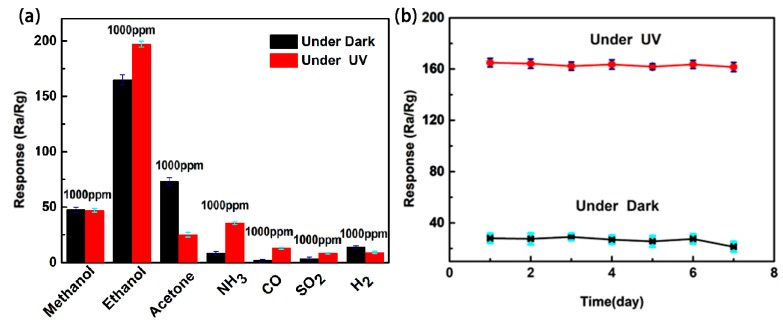
(**a**) Responses to various gases and (**b**) the long-term stability for seven days toward 100 ppm ethanol of the ZnO film sensors under dark and under UV light illumination.

The selectivity study on ZnO film sensors (with and without UV illumination) was performed in the presence of a variety of target gases and the obtained results are displayed in Figure 7a. Sulfur dioxide (SO_2_), methanol (CH_3_OH), ethanol (C_2_H_5_OH), acetone (CH_3_COCH_3_), ammonia (NH_3_), carbon monoxide (CO), and hydrogen (H_2_) were used as target gases. The selectivity study revealed that both the ZnO sensors showing a maximum response to C_2_H_5_OH as compared to other target gases. It is obvious that an appreciable increase in the response of the ZnO sensor with UV light illumination toward C_2_H_5_OH compared to other target gases is noticeably observed, indicating its highest degree of selectivity. The maximum response of 163 to UV-illuminated ZnO film sensor upon 100 ppm exposure of C_2_H_5_OH was noticed. The long-term stabilities of the ZnO film sensor under dark and under UV light illumination were monitored at 100 ppm ethanol for seven days after aging treatment. It can be observed (Figure 7b) that the response varies slightly, indicating good sensor long-term stability.

The obvious improvement of the response to C_2_H_5_OH with UV light illumination at a lower temperature may arise from the photocatalytic conversion of C_2_H_5_OH [23]. The photon-generated electron-hole pairs would activate the chemically-adsorbed oxygen on the surface of ZnO film. Then the C_2_H_5_OH reacts with oxygen species (O^2−^) and produce carbon dioxide and water. This process will release the electrons back to the conductance band of ZnO; as a result, the resistance of the sensor decreases, simultaneously.

(1) $$h\nu \to h^{+}+e^{-}\left(\mathrm{UV}\right)$$

(2) $$O_{2}+e^{-}\left(UV\right)=O_{2}^{-}(ads)$$

(3) $$CH_{3}CH_{2}OH+O_{2}{}^{-}(ads)\;=\;CO_{2}+H_{2}O+2e^{-}$$

We have compared our observed gas sensing results (with UV illumination) with those of reports available in the literature on ZnO and the corresponding comparison is tabulated in Table 1. It is noted that the sensor based on ZnO film in the present study displays a much lower detection limit and higher sensitivity when compared to other ZnO materials reported elsewhere [24,25,26,27,28,29]. The possible reasons of excellent sensing performance of the ZnO film sensor with UV activation are discussed. Firstly, the photocatalytic oxidation of ethanol on the surface of oxide species can improve electron donation to the ZnO film. Secondly, the planar film structure deposited by magnetron sputtering has a high utilizing efficiency of UV light, high electron mobility, and low grain-boundary barrier. Thirdly, a good surface/volume ratio of the ZnO film with a relatively rough and porous surface may promote the gas adsorption and diffusion on the active surface.

## 4. Conclusions

The magnetron sputtering method was successfully employed for the deposition of ZnO film and utilized for the application of ethanol sensors. The as-deposited ZnO film sensor exhibits good sensitivity, low operating temperature, excellent selectivity, and a low detection limit with the activation UV LED. The observed gas sensing results indicate that the as-deposited ZnO film is a promising candidate for applications in the detection of low concentrations of C_2_H_5_OH.

## Figures and Tables

**Table 1:** Comparison of sensing properties of ZnO sensors with different microstructures under UV illumination.

Nanostructure of ZnO	UV Wavelength (nm)/Energy	Working Temperature (°C)	Ethanol (ppm)	Response	Detection Limit (ppm)	Reference
ZnO-SnO_2_ nanoparticles	380/60 mW/cm^2^	250	1000	23 (Ra/Rg)	100	[19]
ZnO nanoparticles	400/2.2 mW/cm^2^	RT	100	1.6 (△I/Ia)	10	[23]
ZnO nanodisk	365/1.6 mW/cm^2^	RT	200	0.3 (△I/Ia)	20	[24]
ZnO nanofiber	365/8 W/cm^2^	RT	60	0.8 (△I/Ia)	10	[25]
ZnO porous film	365/3.6 mW/cm^2^	-	100	1.5 (△I/Ia)	30	[26]
ZnO:AuNPs	254/4.1 mW/cm^2^	125	1000	6.3 (Ra/Rg)	100	[27]
ZnO nanowire	365/100 mW/cm^2^	53	100	1.2 (△R/Ra)	50	[28]
ZnO film	365/0.5 W/cm^2^	170	100	163 (Ra/Rg)	0.1	This work

## Supplementary Materials

Supplementary File 1

## References

[1] Lee S., Bang S., Park J., Park S., Jeong W., Jeon H. (2010). The effect of oxygen remote plasma reatment on ZnO TFTs fabricated by atomic layer deposition. Phys. Status Solidi.

[2] Da Silva L.F., M’Peko J.-C., Catto A.C., Bernardini S., Mastelaro V.R., Aguir K., Ribeiro C., Longo E. (2017). UV-enhanced ozone gas sensing response of ZnO-SnO_2_ heterojunctions at room temperature. Sens. Actuators B Chem..

[3] Joshi N., da Silvac L.F., Jadhav H.S., Shimizu F.M., Suman P.H., M’Peko J.-C., Orlandi M.O., Seo J.G., Mastelaro V.R., Oliveira O.N. (2018). Yolk-shelled ZnCo_2_O_4_ microspheres: Surface properties and gas sensing application. Sens. Actuators B Chem..

[4] Zhu L., Zeng W. (2017). Room-temperature gas sensing of ZnO-based gas sensor: A review. Sens. Actuators A Phys..

[5] Kango S., Kalia S., Celli A., Njuguna J., Habibi Y., Kumar R. (2013). Surface modification of inorganic nanoparticles for development of organic–inorganic nanocomposite—A review. Progress Polym. Sci..

[6] Kant S., Pathania D., Singh P., Dhiman P., Kumar A. (2014). Removal of malachite green and methylene blue by Fe_0.01_Ni_0.01_Zn_0.98_/O Polycrylamide nanocomposite using coupled absorption and Photocatalysis. Appl. Catal. B Environ..

[7] Zhao M.G., Wang X.C., Ning L.L., Jia J.F., Li X.J., Cao L.L. (2011). Electrospun Cu-doped ZnO nanofibers for H_2_S sensing. Sens. Actuators B Chem..

[8] Arnold S.P., Prokes S.K., Perkins F.K., Zaghloul M.E. (2009). Design and performance of a simple room-temperature Ga_2_O_3_ nanowire gas sensor. Appl. Phys. Lett..

[9] Choi Y.J., Hwang I.S., Park J.G., Choi K.J., Park J.H., Lee J.H. (2008). Novel fabrication of an SnO_2_ nanowire gas sensor with high sensitivity. Nanotechnology.

[10] Wu D.Z., Xiao Z.M., Teh K.S., Han Z.B., Luo G.X., Shi C., Sun D.H., Zhao J.B., Lin L.W. (2016). High-throughput rod-induced electrospinning. J. Phys. D Appl. Phys..

[11] Fan S.W., Srivastava A.K., Dravid V.P. (2010). Nanopatterned Poly-crystalline ZnO for room temperature gas sensing. Sens. Actuators B Chem..

[12] Fan S.W., Srivastava A.K., Dravid V.P. (2009). UV-activated room-temperature gas sensing mechanism of polycrystalline ZnO. Appl. Phys. Lett..

[13] Camagni P., Faglia G., Galinetto P., Perego C., Samoggia G., Sberveglieri G. (1996). Photo-sensitivity activation of SnO_2_ thin film gas sensors at room temperature. Sens. Actuators B Chem..

[14] Pradesa J.D., Diaz R.J., Ramirezb F.H., Barth S., Cireraa A., Rodriguez A.R., Mathurc S., Morante J.R. (2009). Equivalence between thermal and room temperature UV light-modulated responses of gas sensors based on individual SnO_2_ nanowires. Sens. Actuators B Chem..

[15] Giberti A., Malagu C., Guidi V. (2012). WO_3_ sensing properties enhanced by UV illumination: an evidence of surface effect. Sens. Actuators B Chem..

[16] Kuang Q., Lao C.S., Li Z., Liu Y.Z., Xie Z.X., Zheng L.S., Wang Z.L. (2008). Enhancing the photon and gas sensing properties of a single SnO_2_ nanowire based nanodevice by nanoparticle surface functionalization. J. Phys. Chem. C.

[17] Wang C.Y., Cimalla V., Kups T., Rohlig C.C., Stauden T., Ambacher O. (2007). Integration of In_2_O_3_ nanoparticle based ozone sensors with GaInN/GaN light emitting diodes. Appl. Phys. Lett..

[18] Shinar R., Zhou Z.Q., Choudhury B., Shinar J. (2006). Structurally integrated organic light emitting device-based sensors for gas phase and dissolved oxygen. Anal. Chim. Acta.

[19] Sun J.B., Liu F.M., Zhong T.G., Xu J., Zhang Y.Q., Lu G.Y. (2011). Effectsof UV light illumination on the gas sensing properties of ZnO–SnO_2_ thick film sensor. Sens. Lett..

[20] Tamvakos A., Calestani D., Tamvakos D., Mosca R., Pullini D., Pruna A. (2015). Effect of grain-size on the ethanol vapor sensing properties of room-temperature sputtered ZnO thin films. Microchim. Acta.

[21] Chen J.T., Yan X.B., Liu W.W., Xue Q.J. (2011). The ethanol sensing property of magnetron sputtered ZnO thin films modified by Ag ion implantation. Sens. Actuators B Chem..

[22] Vijayalakshmi K., Karthick K., Dhivya P., Sridharan M. (2013). Low power deposition of high quality hexagonal ZnO film grown on Al_2_O_3_ (0001) sapphire by dc sputtering. Ceram. Int..

[23] Chen Y., Li X.G., Wang J., Tang Z.A. (2016). UV activated hollow ZnO microspheres for selective ethanol sensors at low temperatures. Sens. Actuators B Chem..

[24] De Lacy Costello B.P.J., Ewen R.J., Ratcliffe N.M., Richards M. (2008). Highly sensitive room temperature sensors based on the UV-LED activation of zinc oxide nanoparticles. Sens. Actuators B Chem..

[25] Alenezi M.R., Alshammari A.S., Jayawardena K.D.G.I., Beliatis M.J., Henley S.J., Silva S.R.P. (2013). Role of the exposed polar facets in the performance of thermally and UV activated ZnO nanostructured gas sensors. J. Phys. Chem. C.

[26] Gong J., Li Y., Chai X., Hu Z., Deng Y. (2010). UV-light-activated ZnO fibers for organic gas sensing at room temperature. J. Phys. Chem. C.

[27] Chen H., Liu Y., Xie C., Wu J., Zeng D., Liao Y. (2012). A comparative study on UV light activated porous TiO_2_ and ZnO film sensors for gas sensing at room temperature. Ceram. Int..

[28] Wongrat E., Chanlek N., Chueaiarrom C., Samransuksamer B., Hongsith N., Choopun S. (2016). Low temperature ethanol response Enhancement of ZnO nanostructures sensor decorated with gold nanoparticles exposed to UV illumination. Sens. Actuators A Phys..

[29] Lin C.H., Chang S.J., Chen W.S., Hsueh T.J. (2016). Transparent ZnO-nanowire-based device for UV light detection and ethanol gas sensing on c-Si solar cell. RSC Adv..

