# Supplementary material for: UV-Enhanced Ethanol Sensing Properties of RF Magnetron-Sputtered ZnO Film
Source: Sensors (Basel). 2017 Dec 26;18(1):50. doi: 10.3390/s18010050 (PMC5795609; doi:10.3390/s18010050)
Supplement: Supplementary File 1 [file sensors-18-00050-s001.pdf]

Supporting information

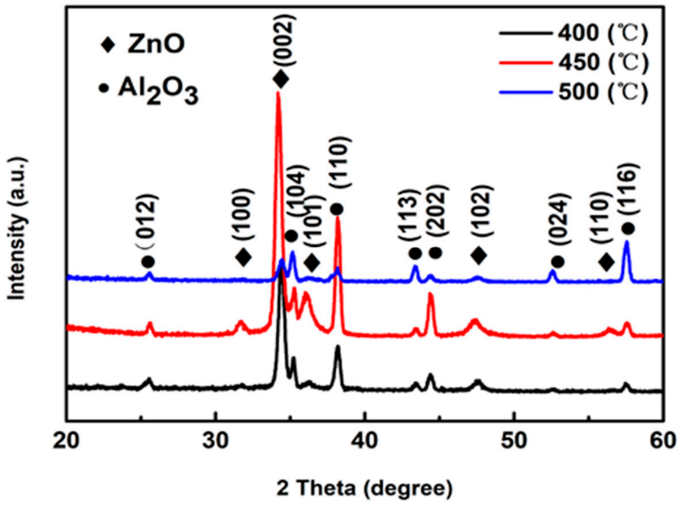

Fig. S1 XRD patterns of the ZnO film annealed at 400 °C, 450 °C and 500 °C, respectively.

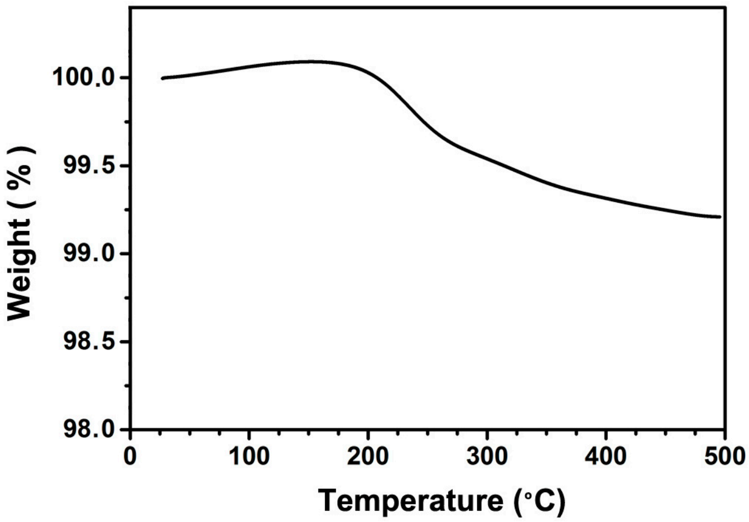

Fig. S2 TGA of the ZnO film from room temperature to 500 °C

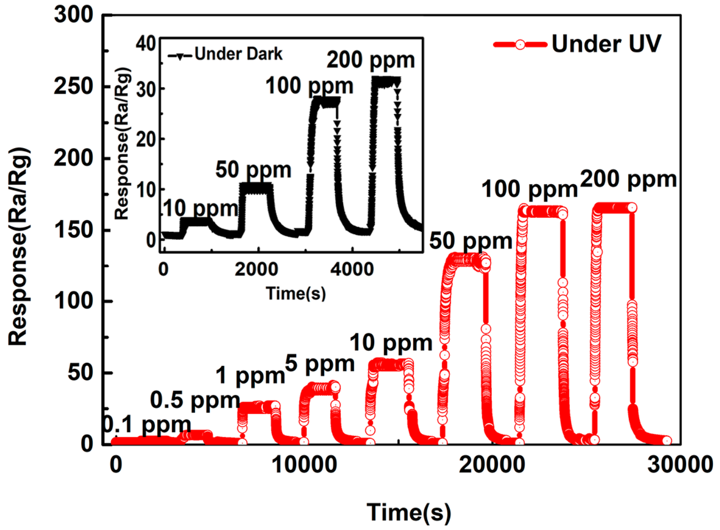

Fig. S3 Response curve of the ZnO film sensor to different concentrations of C_2H_5OH from 0.1 to 200 ppm under dark and under UV LED activation.

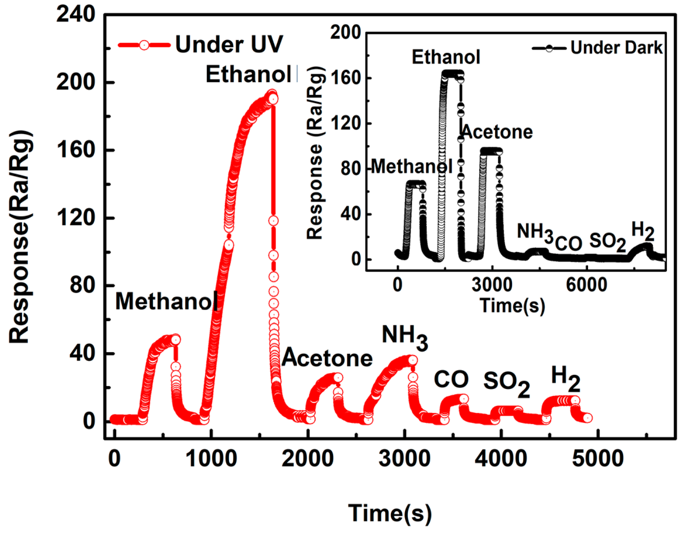

Fig. S4 Responses curve of the ZnO film sensor to various gases of 1000 ppm under dark and under UV light illumination.
